# East African Trypanosomiasis in a Pregnant Traveler

**DOI:** 10.3201/eid1511.090384

**Published:** 2009-11

**Authors:** Behzad Nadjm, Chris Van Tulleken, Douglas Macdonald, Peter L. Chiodini

**Affiliations:** The Hospital for Tropical Diseases, London, UK (B. Nadjm, C. Van Tulleken, P.L. Chiodini); London School of Hygiene and Tropical Medicine, London (P.L. Chiodini, B. Nadjm); Chelsea and Westminster Hospital, London (D. Macdonald)

**Keywords:** Human African trypanosomiasis, Africa, pregnancy, parasites, travelers, letter

**To the Editor**: Human African trypanosomiasis (HAT) results in a large number of deaths and considerable illness in sub-Saharan Africa. Although the disease is uncommon in returning travelers from that region, awareness of it is important for medical practitioners in areas where the disease is not endemic. The disease can be categorized geographically into West and East African trypanosomiasis caused by *Trypanosoma brucei gambiense* and *T. brucei rhodesiense*, respectively, and clinicopathologically into hemolymphatic (stage I) disease and meningoencephalitic (stage II) disease (*1*). The East African form of the disease is less common than the West African form and accounts for 10% of the global incidence of trypanosomiasis.

Relative stability in East African nations may have contributed to the lower incidence of the disease in these countries, but drought and increasing pressure on water sources may lead to an upsurge in East African disease. The increasing ease of global travel and attraction of game safaris and hunting may also lead to increasing exposure in travelers. HAT is treated with toxic drugs in regimens that have changed little for decades. Few published data exist on the treatment of HAT in pregnancy, particularly for East African disease. We describe a case of *T. brucei rhodesiense* infection occurring in a pregnant traveler.

A 32-year-old woman, 20 weeks pregnant, returned from a 9-day safari trip to Tanzania 8 days before coming to a hospital in London. She described a short history of fever, headache, and soft-tissue swelling of the forehead with severe regional adenopathy. She had evidence of skin necrosis (chancre) (Figure) but no history of tsetse fly bite. Blood tests showed anemia (hemoglobin 9.5 g/dL), leukopenia (1.8 × 10^9^ cells/L), and thrombocytopenia (60 × 10^9^ cells/L). A blood film showed trypomastigotes of *T. brucei rhodesiense*. Suramin was initially unavailable for treatment, but because of her deteriorating clinical state, she was treated with 1 dose of pentamidine (4 mg/kg) before suramin was obtained. Suramin was begun 36 hours after admission, initially at 5 mg/kg and increased over the next 2 doses up to 1 g. During the next 48 hours, her fever resolved, and serial blood films showed clearance of the parasites from the blood. A cerebrospinal fluid sample showed no signs of stage II disease, and the patient continued on suramin, completing a standard course as an outpatient. Her pregnancy was closely monitored, and she gave birth at term to a healthy baby girl.

**Figure:**
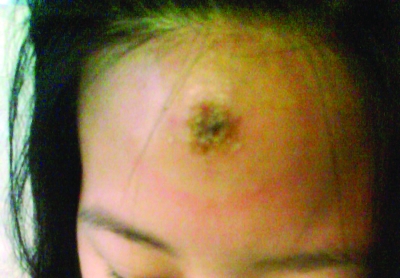
Chancre at site of tsetse fly bite on forehead of pregnant patient with trypanosomiasis.

The treatment of choice for stage I HAT caused by *T. brucei gambiense* is parenteral pentamidine (*1*). No published trials compare pentamidine and suramin in East African trypanosomiasis, but longstanding consensus suggests that suramin is more likely to be efficacious in stage I East African disease (*1*). The basis for this difference in efficacy is unexplained.

Theoretically, pentamidine may be teratogenic because it inhibits protein and nucleic acid synthesis in vitro (*2*). However, studies in rats found pentamidine to be feticidal but not teratogenic (*3*). Pentamidine has been used extensively for HAT prophylaxis without reported problems (*4*) and has had limited use in pregnant women with *Pneumocystis* *jirovecii* pneumonia. It continues to be recommended in pregnant women with stage I HAT originating in West Africa. Suramin is known to cause a syndrome similar to preeclampsia in pregnant rats (*5*), yet it too has been used in large-scale treatment programs for onchocerciasis, and no fetal or placental effects have been reported in humans (*2**,**6*). We found 1 case report describing successful use of suramin, followed by melarsoprol, in a pregnant woman with HAT (*7*).

The treatment of stage II disease in pregnancy is problematic, and published information to guide therapy is lacking. Although the effect of arsenicals on fetuses is a concern, case reports have described the successful use of melarsoprol during pregnancy (*7**,**8*); if left untreated, the disease is fatal. Thus, if our patient had stage II disease, use of melarsoprol, which is often given with prednisolone, would have been necessary.

In pregnant women with West African (*T. brucei gambiense*) stage II disease, either melarsoprol or eflornithine can be used, but neither is effective for East African disease. Although eflornithine can abort early pregnancies and cause disordered organogenesis (*9*), the severe encephalopathy associated with melarsoprol makes eflornithine a preferable option for single-agent treatment. However, nifurtimox–eflornithine combination therapy will soon replace single-drug regimens for stage II *T. brucei gambiense* cases (*10*).

We believed evidence was insufficient to withhold suramin therapy for this highly fatal disease. Because of the uncertainty about effects of pregnancy on the ability to clear trypanosomes, the patient will be followed up for signs of relapse. The danger of HAT should be specifically highlighted for all travelers to trypanasomiasis-endemic regions, particularly pregnant travelers because of potential harm to unborn children.
